# Population structure and gene flow in the Sheepnose mussel (*Plethobasus cyphyus*) and their implications for conservation

**DOI:** 10.1002/ece3.8630

**Published:** 2022-02-17

**Authors:** Sara Schwarz, Kevin J. Roe

**Affiliations:** ^1^ Department of Natural Resources Ecology and Management Iowa State University Ames Iowa USA; ^2^ 1177 Ecology and Evolutionary Biology Program Iowa State University Ames Iowa USA

**Keywords:** genetic diversity, microsatellite, migration, mtDNA, population genetics, Unionidae

## Abstract

North American freshwater mussel species have experienced substantial range fragmentation and population reductions. These impacts have the potential to reduce genetic connectivity among populations and increase the risk of losing genetic diversity. Thirteen microsatellite loci and an 883 bp fragment of the mitochondrial ND1 gene were used to assess genetic diversity, population structure, contemporary migration rates, and population size changes across the range of the Sheepnose mussel (*Plethobasus cyphyus*). Population structure analyses reveal five populations, three in the Upper Mississippi River Basin and two in the Ohio River Basin. Sampling locations exhibit a high degree of genetic diversity and contemporary migration estimates indicate that migration within river basins is occurring, although at low rates, but there is no migration is occurring between the Ohio and Mississippi river basins. No evidence of bottlenecks was detected, and almost all locations exhibited the signature of population expansion. Our results indicate that although anthropogenic activity has altered the landscape across the range of the Sheepnose, these activities have yet to be reflected in losses of genetic diversity. Efforts to conserve Sheepnose populations should focus on maintaining existing habitats and fostering genetic connectivity between extant demes to conserve remaining genetic diversity for future viable populations.

## Graphical abstract

Population genetic analyses of the Sheepnose mussel reveal five populations. Sampling locations exhibit a high degree of genetic diversity and contemporary migration estimates indicate that migration between populations within river basins is occurring, although at low rates. But no migration is occurring between the Ohio and Mississippi river basins.

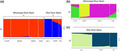

## 1 INTRODUCTION

Freshwater mussels in the family Unionidae, are considered one of the most imperiled taxa in the world and a substantial proportion of freshwater mussel diversity can be found in North America (Bogan, 2008; Graf & Cummings, 2007; Haag & Williams, 2014). North America is home to about 297 native freshwater mussel species and ~70% of these species are considered endangered, threatened, or of special concern with only 24% of the species considered to be stable (Williams et al., 2011). Humans have indirectly impacted critical habitat through climate change effects (Dudgeon et al., 2005; Haag & Williams, 2014; Strayer & Dudgeon, 2010; Williams et al., 2011) and directly impacted populations by constructing dams, channelizing, and polluting rivers. (Williams et al., 1989). Mussel species declines are an increasing concern because of their crucial role providing ecosystem services such as environmental nutrient recycling, structural habitat for other species, food resource, and biofiltration (USFWS, 2012a; Vaughn, 2018; Vaughn et al., 2008). Their ecosystem services and intrinsic value warrant the development of comprehensive conservation strategies to preserve them. To achieve this, researchers have started to examine the ecological and genetic conditions of imperiled freshwater mussel species at the population level. Successful conservation of imperiled species, such as freshwater mussels, must include a measurement of available genetic diversity as it represents the raw material for adaptation to environmental changes. Lack of genetic diversity can cause populations to become genetically fixed and intolerant to a constantly changing environment (Frankel & Soulé, 1981). Population connectivity is important for maintaining genetic diversity and can be heavily impacted by anthropogenic activity. In this manuscript, we estimate the distribution of genetic diversity and population connectivity to inform conservation decisions of an imperiled freshwater mussel species, the Sheepnose mussel (*Plethobasus cyphyus*).

There are three species within the genus *Plethobasus*, all of which are listed as endangered. Of the three species, the Sheepnose currently occupies the broadest distribution (Hove et al., 2015; Turgeon et al., 1998. All three species of *Plethobasus* have exhibited range‐wide population declines presumably as a result of anthropogenic changes to their habitat and it is thought that these changes are the most prominent factor in the decline of *Plethobasus* (Stein & Flack, 1997; USFWS Service, 1984; US ACOE, 2011). Unlike its much rarer congeners, the relatively high abundance and widespread nature of the Sheepnose allows for an opportunity to conserve this species.

Sheepnose usually occur in shallow shoals with moderate‐to‐swift currents over coarse gravel and sand (Oesch, 1984). However, other habitat features may include mud, cobble, and boulders in deeper large river runs (Parmalee & Bogan, 1998). In general, freshwater mussels are long‐lived species with life spans ranging from two years to decades (Mutvei et al., 1994). Sheepnose are estimated to live ~20–30 years and become reproductively mature around 5 years of age (Hove et al., 2015). The Sheepnose, as well as all unionid species, utilize a mating strategy in which males expel sperm into the water column, which are then taken in by females for fertilization of their eggs which are held in modified portions of their gills. After fertilization, the mature larvae, glochidia, are released into the water and attach to the gills or fins of fish where they complete their development. After development is complete, the juvenile mussels then drop off the gills and establish themselves in the substrate (Parmalee & Bogan, 1998). Originally, the only known natural host fish for the Sheepnose was the Sauger (*Sanders candensis*) (Surber, 1913), however, a more recent study has shown that the Sheepnose appears to be a cyprinid host specialist (Hove et al., 2015). Gene flow is thought to be achieved through the dispersal of sperm and the glochidia larval stage (Ferguson et al., 2013; Hove et al., 2015) so conservation efforts must include consideration of host species availability so the life cycle can remain complete.

**FIGURE 1:**
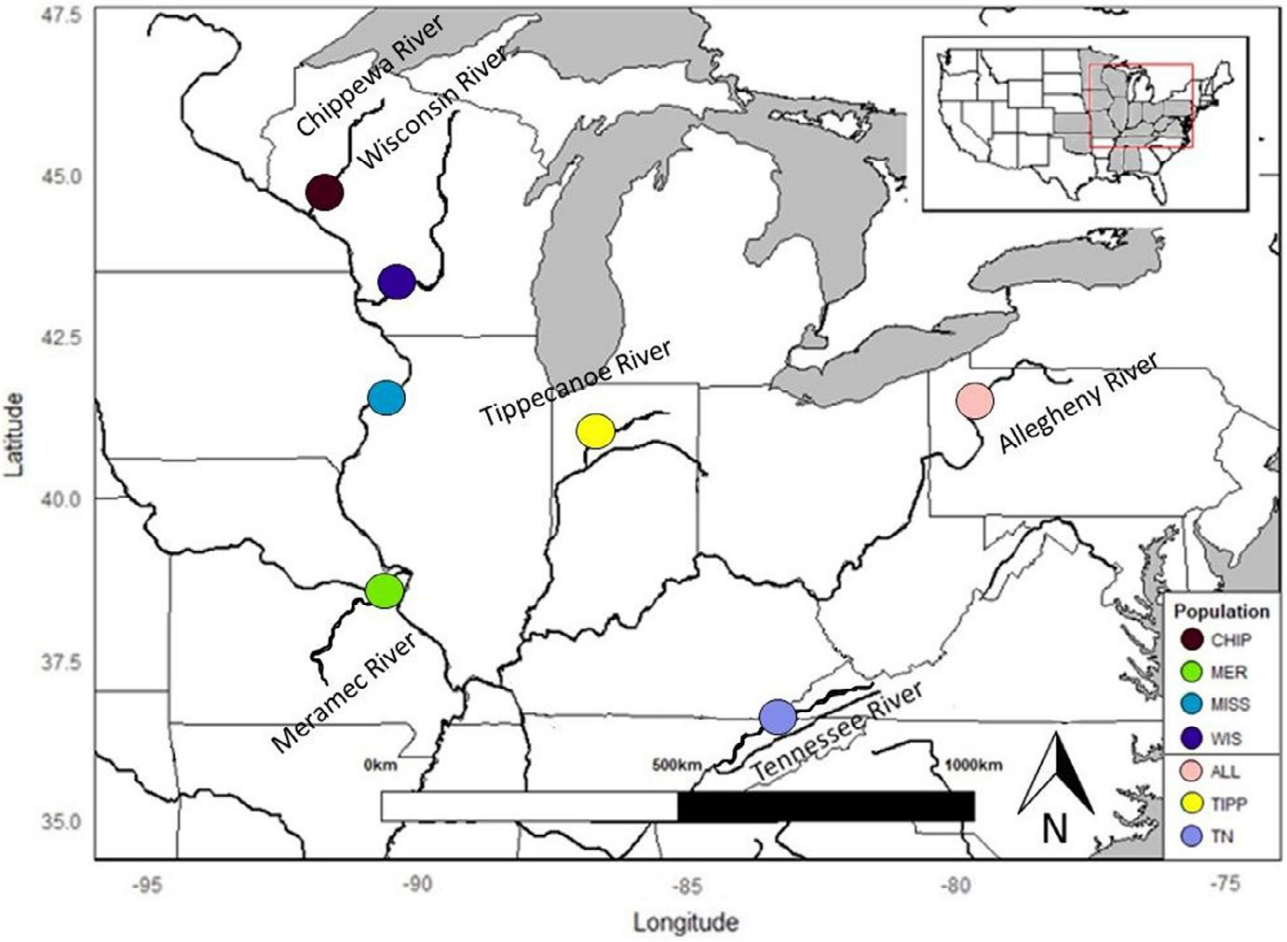
Sampling locations of the seven sites from which Sheepnose mussels were collected for genetic analysis. The gray shaded area in the inset map indicates the approximate historic range of the Sheepnose (NatureServe Explorer)

Historically, the Sheepnose mussel occurred throughout much of the Mississippi River system (Figure 1), including The Ohio River Basin (Allegheny, Kanawah, Ohio, Tennessee, Tippecanoe rivers), Mississippi River Basin (Big Sunflower, Chippewa, Gasconade, Meramec, St. Croix, Wisconsin rivers) (USFWS, 2002). However, as of April 2012, the Sheepnose was listed as endangered under the Endangered Species Act (USFWS, 2012b), primarily because it had been extirpated from two‐thirds of its former range and remaining locations appear to be spatially and genetically isolated from each other (USFWS, 2002). According to a status report conducted in 2002 by the USFWS, of the 77 streams that were historically occupied by Sheepnose populations, only 26 streams are thought to still be occupied. This decline in range and abundance has been attributed to human impacts such as land development, dams, and pollution (Haag & Williams, 2014). However, published and unpublished records since the 1800s, indicate that although Sheepnose was historically widespread, the species was often described as uncommon (USFWS, 2002). Archaeological evidence of discovered shell fragment locations indicates that this species may have been uncommon or rare for centuries (Parmalee & Bogan, 1998). The goals of this study are to describe the genetic diversity and population structure across the majority of the extant range of the Sheepnose. The loss of Sheepnose populations across its range indicates the possibility that a loss of genetic diversity and population connectivity has also occurred. Under this scenario, populations would possibly show the signature of genetic bottlenecks. If historical evidence is true and the Sheepnose has always been rare, analysis of population genetic data would not show signs of a loss of diversity or evidence of a bottleneck, and extant populations would be more resilient to the effects of isolation. Conservation implications will differ depending on the level of current connectivity and genetic diversity available within populations. If the isolated nature of the Sheepnose populations has resulted in the erosion of genetic diversity, then reestablishing habitat connectivity and implementing translocations could be prudent. If, however, the Sheepnose appears to have maintained genetic diversity despite population loss and isolation, then maintaining available habitat and improving connectivity would likely be a higher priority management objective. This study is intended to provide knowledge about the extent of isolation and genetic diversity of the Sheepnose by estimating contemporary population connectivity and structure to inform conservation decisions.

## 2 MATERIALS AND METHODS

### 2.1 Data collection

#### 2.1.1 Study area and sampling

A combination of microsatellite markers and mitochondrial DNA sequences was used to estimate genetic diversity, population structure, contemporary migration, and population size changes in the Sheepnose. Samples (*N* = 164) for DNA extraction were collected from seven different localities (Table 1). Collection efforts were focused on the Mississippi and Ohio river basins (Figure 1). Mussels were collected by snorkeling or SCUBA at various locations. Samples for DNA extraction were collected by taking a small (~1 mm) biopsy of mantle tissue (Berg et al., 1995) or by using cytology brushes that were swabbed over the mantle tissue of mussels to accumulate mucous and sloughed cells (Henley et al., 2006). Biopsy samples were stored in 95% ethanol and DNA was extracted from mantle tissue samples using the Qiagen DNeasy^®^ Blood and Tissue Kit (Qiagen # 69506) according to the kit instructions. Cytology brush samples were stored in the lysis buffer provided with the Puregene Buccal Cell Core Kit B (Qiagen) and DNA was extracted following the kit instructions. Extracted DNA was quantified using a Nanodrop ND1000 spectrophotometer and stored at 4°C.

**TABLE 1:** Numbers of Sheepnose mussels sampled from seven study sites for microsatellite and mitochondrial genotyping

Site	Site ID	Sample Size	*H*_o_	*H*_E_	Allelic Richness	Private Alleles	*F*
Allegheny	ALL	22	0.772	0.768	7.031	6	0.072
Chippewa	CHIP	21	0.818	0.813	7.359	3	−0.012
Meramec	MER	26	0.834	0.844	8.440	11	0.010
Mississippi	MISS	51	0.825	0.859	8.180	18	0.040
Tippecanoe	TIPP	19	0.860	0.834	8.768	18	−0.017
Tennessee	TN	8	0.837	0.796	8.615	4	−0.052
Wisconsin	WIS	17	0.831	0.836	8.106	17	0.008
Total/Avg	7	164	0.825	0.821	8.071	11	0.007

Note

Results from analysis of microsatellite data. Observed (*H*_o_) and expected (*H*_E_) heterozygosity, allelic richness, private alleles, and fixation index (inbreeding) of each site across all loci.

#### 2.1.2 Microsatellites

Sixteen species‐specific polymorphic microsatellite loci were used to genotype samples. These markers were developed by Genetic Identification Services, Chatsworth, CA (Appendix S1). Polymerase chain reactions (PCR) were performed using 10 µL reactions (~2 ng of genomic DNA was used in each reaction). The standard M13 protocol (Schuelke, 2000) was used with the florescent dye labeled with HEX (Applied Biosystems). Reagents for a 10 µl reaction included: 6.6 µl H_2_0, 1 µl Biolase Buffer (10×), 0.3 µl MgCl_2_, 0.8 µl dNTP, 0.1 µl forward primer, 0.1 µl reverse primer, 0.05 µl M13 labeled oligo, 0.05 µl Biolase Taq polymerase, and 1 µl template DNA. Reactions were run in Eppendorf Master Cycler thermal cyclers under consistent conditions (95°C/5 min; [94°C/30 s; 62°C/60 s; 72°C/30 s] × 10 cycles; [94°C/30 s; 55–59.4 °C/60 s; 72°C/30 s; 72°C/20 min] × 25 cycles; 72°C/4 min). Negative controls were performed with every reaction to detect potential contamination. PCR products were visualized on 1% agarose gels to confirm that the reaction was successful and that the negative control showed no contamination. PCR products were then sent to the Iowa State DNA Facility where they underwent capillary electrophoresis to determine allele sizes. The raw data were then scored using GeneMarker^®^ v1.85 (Hulce et al., 2011) and converted into the desired software input format using base RStudio (RStudio Team, 2016).

#### 2.1.3 Mitochondrial sequences

Mitochondrial DNA sequences of an 883 base pair fragment of the first subunit of the NADH dehydrogenase gene (ND1) were also generated for samples. DNA sequence data for the ND1 gene were generated through PCR with Leu‐uurF (5^′^‐TGGCAGAAAAGTGCATCAGATTAAAGC‐3^′^) and LoGlyR (5^′^‐CCTGCTTGGAAGGCAAGTGTACT‐3^′^) primers (Serb et al., 2003). For a 25 µl reaction: 1 µl LEU UURF primer, 1 µl LoGlyR primer, 9.5 µl H_2_0, 12.5 µl MyTaq polymerase (Bioline), and 1 µl template DNA. Approximately ~1 ng of genomic DNA was used for the template. Reactions were run in Eppendorf Master Cycler thermal cyclers at the following conditions: (95°C/60 s; [95°C/30 s; 50°C/60 s; 72°C/30 s] × 37 cycles; 72°C/60 s; 72°C/60 s). Negative controls were performed with every reaction to detect potential contamination. Products were then run on 1% agarose gel to assure that the reaction was successful and to verify that the negative control showed no contamination. Successful PCR products were prepared for sequencing using the ExoSAP‐IT (US Biochemicals #78250). Template consisted of ~1 µl of genomic DNA in conjunction with forward and reverse sequencing reactions using Big Dye^®^ ver. 3.1 cycle sequencing kit (Applied Biosystems # 4337454) and sent to the Iowa State DNA Facility for sequencing. The raw results were then analyzed and edited using GENEIOUS v8.1.6 (Kearse et al., 2012). Results were also converted into amino acids to confirm that sequences were aligned properly before exporting the matrix for further analyses. DNA sequence data have been submitted to GenBank (Accession Number: MH853483).

### 2.2 Data analysis

#### 2.2.1 Microsatellites

##### Genetic diversity

Each locus was tested for deviations from Hardy–Weinberg expectations, potential genotyping errors, and linkage disequilibrium at each collection site using MICROCHECKER (Van Oosterhout et al., 2004). The average number of alleles (*A*), expected heterozygosity (*H*_e_), observed heterozygosity (*H*_o_), and fixation index (*F*_ST_) were calculated using GenAlEx v6.5 (Peakall & Smouse, 2012). Rarified allelic richness was calculated using the package hierfstat (Goudet & Thibaut, 2015) in RStudio. Private alleles were also determined by site using the package poppr (Kamvar et al., 2014) in RStudio. Pairwise *F*_ST_ (Wright, 1969) values were calculated using GenAlEx. Adjusted *F*_ST_ (*G*’_ST_) (Hedrick, 2005) and Jost's *D* (Jost, 2008) values were calculated using the package DEMEtics (Gerlach et al., 2010) in RStudio in order to account for a potential depression of the standard *F*_ST_ measure due to high allelic diversity (many loci had between 15 and 45 alleles). A permutation test was performed on the *F*_ST_ values using base RStudio to determine degree of genetic differentiation found among sampling locations that were located within versus between drainage basins. This test was permuted 100,000 times and *p*‐values were assessed at a.05 significance level. Microsatellite genotypes have been submitted to DRYAD (https://doi.org/10.5061/dryad.gxd2547mm).

##### Population structure

Clustering of Sheepnose collection sites into distinct genetic groups was conducted using the program STRUCTURE v2.3.4 (Pritchard et al., 2000). STRUCTURE analyses consisted of a burn‐in of 100,000 Markov chain Monte Carlo (MCMC) iterations followed by 1,000,000 iterations using the admixture model and correlated allele frequencies. Each run had 1–8 possible K values (n collection sites +1) and 10 replicates of each run. After an initial analysis detected two populations (*K* = 2), subsequent analyses to detect substructure were also conducted. STRUCTURE runs were conducted on the two populations identified in the initial run using the same procedures as previously, but with possible *K* values of 1–4 and 1–5. The web application POPHELPER (Francis, 2016) was used to determine the most probable value of K utilizing the Evanno method (Evanno et al., 2005) to determine the second‐order rate of change in the distribution of L(K). POPHELPER was also used to merge the 10 replicates together and to graphically display results. An analysis of molecular variance (AMOVA) with 999 permutations was conducted using GenAlEx to further examine Sheepnose population structure.

##### Estimation of migration

The program BAYESASS (Wilson & Rannala, 2003) was used to estimate asymmetrical migration rates. Rates were estimated among collection sites because of the high degree of genetic differentiation observed between sites based on the *F*_ST_ values. BAYESASS estimates genetic flow among sites as a migration rate (*m*) which can be interpreted as the fraction of migrants per generation in one population that is derived from a source population. These estimations are calculated using a Bayesian approach and MCMC sampling to generate values for *m* over the last few (<5) generations (Wilson & Rannala, 2003). Given a generation time of approximately 5 years for the Sheepnose (Hove et al., 2015), BAYESASS estimated *m* values over the past ~25 years. Run lengths and parameters were optimized to ensure convergence and delta parameters were adjusted to accommodate 40–60% acceptance. BAYESASS appeared to reach convergence after five runs with a different initial seed and a Bayesian deviance metric (Spiegelhalter, 2002) was used to select the run that best fit the dataset. TRACER v1.7 (Rambaut et al., 2018) was also used to visualize mixing, suitable burn‐in values, and convergence problems. The final run consisted of the parameters from the best selected run with a run length of 5x10^7^ iterations and sampling every 100 iterations. The burn‐in period consisted of 2x10^7^ iterations.

##### Changes in population size

Considering apparent declines in the number of Sheepnose populations and the potential genetic bottlenecks associated with habitat fragmentation and isolation (Andersen et al., 2004), a test for genetic bottlenecks at each site was conducted using BOTTLENECK (Piry et al., 1999). Both tests implemented by BOTTLENECK were used for the analysis. A Wilcoxon's sign rank test was conducted to determine whether the heterozygosity of a site was less than predicted under mutation‐drift equilibrium. This test can detect bottlenecks over the last 2–4N_e_ generations. The second test uses a mode‐shift test of allele proportions over the last few dozen generations (Cornuet & Luikart, 1996; Luikart et al., 1998). This analysis was conducted with 10,000 replications under the stepwise mutation (SMM) and two‐phase model (TPM) that included 95% single‐step mutations and 5% multi‐step mutations and a variance of 12 as recommended by Piry et al. (1999). All collection sites were tested separately for a bottleneck and the p‐values estimated by the Wilcoxon's sign rank test were assessed at a 0.05 significance level.

#### 2.2.2 Mitochondrial sequences

##### Sequence diversity

Of the 164 Sheepnose samples, 157 were amplified, successfully sequenced and analyzed. Haplotypes were created with DnaSP v6 (Librado & Rozas, 2009). ARLEQUIN v3.5 (Excoffier & Lischer, 2010) was used to estimate haplotype (*H*_d_) and nucleotide (π) diversity for each sampling location. Pairwise *F*_ST_ values among sampling sites were calculated using ARELQUIN and tested for significance using 3000 permutations and a significance level of 0.05. A permutation test was performed on the *F*_ST_ values using base RStudio to determine degree of genetic differentiation found among sites that were located within versus between drainage basins. This test was permuted 100,000 times and *p*‐values were assessed at a 0.05 significance level. PopART (Leigh & Bryant, 2015) was used to create a minimum spanning network (Bandelt et al., 1999) of all haplotypes.

##### Population expansion

A mismatch distribution analysis using ARLEQUIN was performed on all sampling sites. This analysis estimates pairwise differences among all the sequences. A population that has not changed in effective size over a long period of time will display a ragged distribution of pairwise distances, whereas a population that has been growing generates distributions that are smoother (Harpending, 1994). A raggedness index is estimated based on this distribution and can be assessed to interpret whether population expansion has occurred (Harpending et al., 1993). Estimates of demographic expansion were generated over 1,000 bootstrap replicates. *p*‐values for the raggedness index and sum of square deviations (SSD) were assessed at a 0.05 significance level.

## 3 RESULTS

### 3.1 Microsatellites

#### 3.1.1 Genetic diversity

Two of the microsatellite loci (C109A and C115) amplified poorly and were subsequently dropped from the analysis. Only one locus (D113) exhibited an excess of homozygosity and potential null alleles at multiple sampling locations, and it was also dropped from analysis. Microsatellite analysis results described below are from the remaining 13 microsatellite loci (Appendix S1). MICROCHECKER analysis revealed that 5 loci were out of Hardy–Weinberg proportions at 1–2 sampling sites. There were 295 alleles across the 13 loci with 11 to 47 alleles per locus (Appendix S1). Observed heterozygosity was high (>0.70) at all sites (Table 1). Allelic richness averaged 8.071 alleles over all sampling locations. The number of private alleles ranged from 3 (CHIP) to 18 (MISS and TIPP) (Table 1). The fixation index (*F*) ranged from −0.052 at the TN site to 0.072 at the ALL site (Table 1). Standard *F*_ST_ values (Wright, 1969), *G*’_ST_, and Jost's *D* metrics were also calculated. Since the standard *F*_ST_ values were likely depressed due to the high number of alleles at several loci, only *G*’_ST_ and Jost's *D* values were reported (Table 2). The *G*’_ST_ values indicated a high degree of population differentiation at all sampling sites except MISS and WIS (0.0623). The permutation test indicated a significant difference (*p* = .005) between samples found in the Upper Mississippi River Basin (CHIP, MISS, MER, and WIS) and the Ohio River Basin (ALL, TIPP, and TN).

**TABLE 2:** Pairwise adjusted *G*’_ST_ (below diagonal) and Jost's *D* (above diagonal) values for microsatellite data among all sites

	ALL	CHIP	MER	MISS	TIPP	TN	WIS
ALL		0.3680	0.5726	0.3688	0.1047	0.5703	0.4150
CHIP	0.4168		0.2062	0.1011	0.2539	0.4535	0.0533
MER	0.6181	0.2300		0.1868	0.4336	0.4674	0.1610
MISS	0.4122	0.1114	0.2045		0.2820	0.3827	0.0580
TIPP	0.1223	0.2831	0.4679	0.3086		0.4129	0.3183
TN	0.6190	0.4953	0.5048	0.4174	0.4504		0.4199
WIS	0.4621	0.0587	0.1784	0.0623	0.3495	0.4578	

#### 3.1.2 Population structure

**FIGURE 2:**
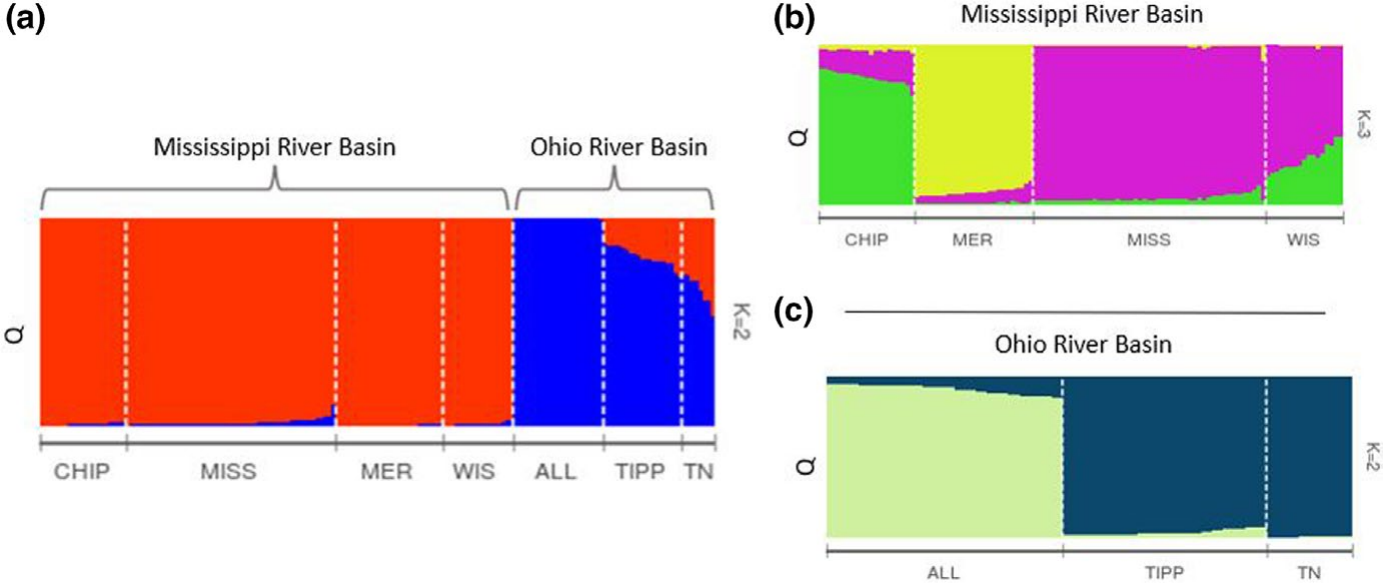
(a) STRUCTURE clustering of the seven sampled Sheepnose populations into *K* = 2 groups corresponding to Upper Mississippi (CHIP, MER, MISS, and WIS) and Ohio River (ALL, TIPP, and TN) basins. (b) The Mississippi River Basin group clustered into *K* = 3 groups with CHIP and WIS clustering together and MER and MISS clustering on their own. (c) Ohio River Basin group clustered into *K* = 2 with TIPP and TN clustering together and ALL clustering on its own

The STRUCTURE analysis including all sampling locations indicated that the most likely value of *K* was 2 (Evanno et al., 2005). Sampling sites in the Upper Mississippi River Basin (CHIP, MER, MISS, and WIS) strongly clustered together and represent a different cluster from sites in the Ohio River Basin (ALL, TIPP, and TN) (Figure 2a). An AMOVA analysis also indicated significant genetic differentiation between the Mississippi and Ohio river basins (*p* ≤ .0001). Despite sampling sites clustering into major river basins, with few exceptions, each sampling location showed a high degree of differentiation from other sites (Table 2). Because the sample sites exhibited substantial genetic differentiation, and populations may be structured hierarchically, with coarser structure obscuring more fine‐scale structure, additional STRUCTURE analyses were conducted separately on the two initial clusters. STRUCTURE analysis of the Upper Mississippi River Basin indicated a *K* value of 3 (Figure 2b), clustering the MER and CHIP sites by themselves and the WIS, and MISS sites clustered together. Analysis of the Ohio River Basin indicated a *K* value of 2 with the ALL site clustered separately from the TIPP and TN sites (Figure 2c).

#### 3.1.3 Estimation of migration

**FIGURE 3:**
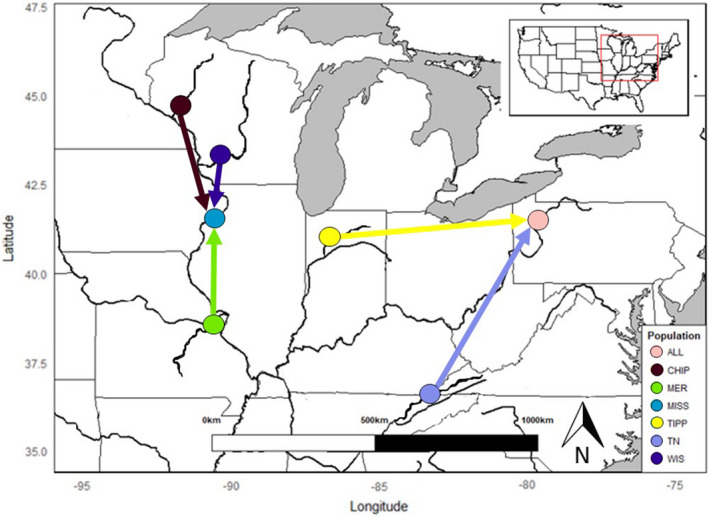
Pattern of contemporary gene flow estimated by BAYESASS (Wilson & Rannala, 2003) between sampling sites that had values greater than 0.1. The arrow colors and arrowheads indicate the direction of migration from the sources

Contemporary migration rates estimated by BAYESASS indicated that migration between most sample sites was low. Only five of the 42 pairwise comparisons exhibited rates greater than 0.1 (Table 3). The permutation test indicated that there was significantly (*p* = .008) more migration occurring among sampling sites within the same drainage basin (Mississippi and Ohio basins) than sites found in different drainage basins. All instances of migration rates >0.1 were asymmetrical, with migrants moving in only one direction. Three of these were in the Upper Mississippi River Basin and two were in the Ohio River Basin (Table 3, Figure 3).

**TABLE 3:** Asymmetrical pairwise contemporary migration rates and associated 95% confidence intervals generated by BAYESASS

	ALL	CHIP	MER	MISS	TIPP	TN	WIS
ALL		0.012	0.012	0.012	0.012	0.011	0.012
C.I. (95%)		(−0.010, 0.033)	(−0.0010, 0.033)	(−0.010, 0.034)	(−0.011, 0.034)	(−0.010, 0.033)	(−0.011, 0.034)
CHIP	0.012		0.0119	0.260*	0.012	0.012	0.012
C.I. (95%)	(−0.011, 0.034)		(−0.010, 0.034)	(0.240, 0.284)	(−0.038, 0.062)	(−0.011, 0.035)	(−0.011, 0.034)
MER	0.011	0.010		0.245*	0.010	0.010	0.0101
C.I. (95%)	(−0.010, 0.032)	(−0.009, 0.029)		(0.196, 0.294)	(−0.050, 0.071)	(−0.009, 0.029)	(−0.009, 0.029)
MISS	0.019	0.006	0.016		0.006	0.0057	0.006
C.I. (95%)	(−0.006, 0.044)	(−0.005, 0.017)	(−0.005, 0.027)		(−0.032, 0.043)	(−0.005, 0.017)	(−0.005, 0.017)
TIPP	0.255*	0.013	0.013	0.015		0.013	0.013
C.I. (95%)	(0.200, 0.310)	(−0.011, 0.037)	(−0.011, 0.037)	(−0.009, 0.039)		(−0.011, 0.037)	(−0.011, 0.037)
TN	0.174*	0.022	0.034	0.036	0.022		0.022
C.I. (95%)	(0.087, 0.260)	(−0.019, 0.063)	(−0.006, 0.075)	(−0.018, 0.091)	(−0.039, 0.083)		(−0.019, 0.063)
WIS	0.014	0.014	0.016	0.248*	0.014	0.014	
C.I. (95%)	(−0.012, 0.040)	(−0.012, 0.040)	(−0.010, 0.042)	(0.218, 0.278)	(−0.044, 0.071)	(−0.012, 0.040)	

Note

The left column represents the source site and the top row represents the receiving sites.

*Indicates estimated rates >0.1.

#### 3.1.4 Changes in population size

No significant values indicating bottlenecks were obtained based on the Wilcoxon's Sign Test. All sampling sites also exhibited an L‐shaped distribution characterized by a high proportion of low‐frequency alleles and a smaller proportion of alleles of intermediate frequencies indicating no recent bottlenecks had occurred.

### 3.2 Mitochondrial sequences

#### 3.2.1 Sequence diversity

**FIGURE 4:**
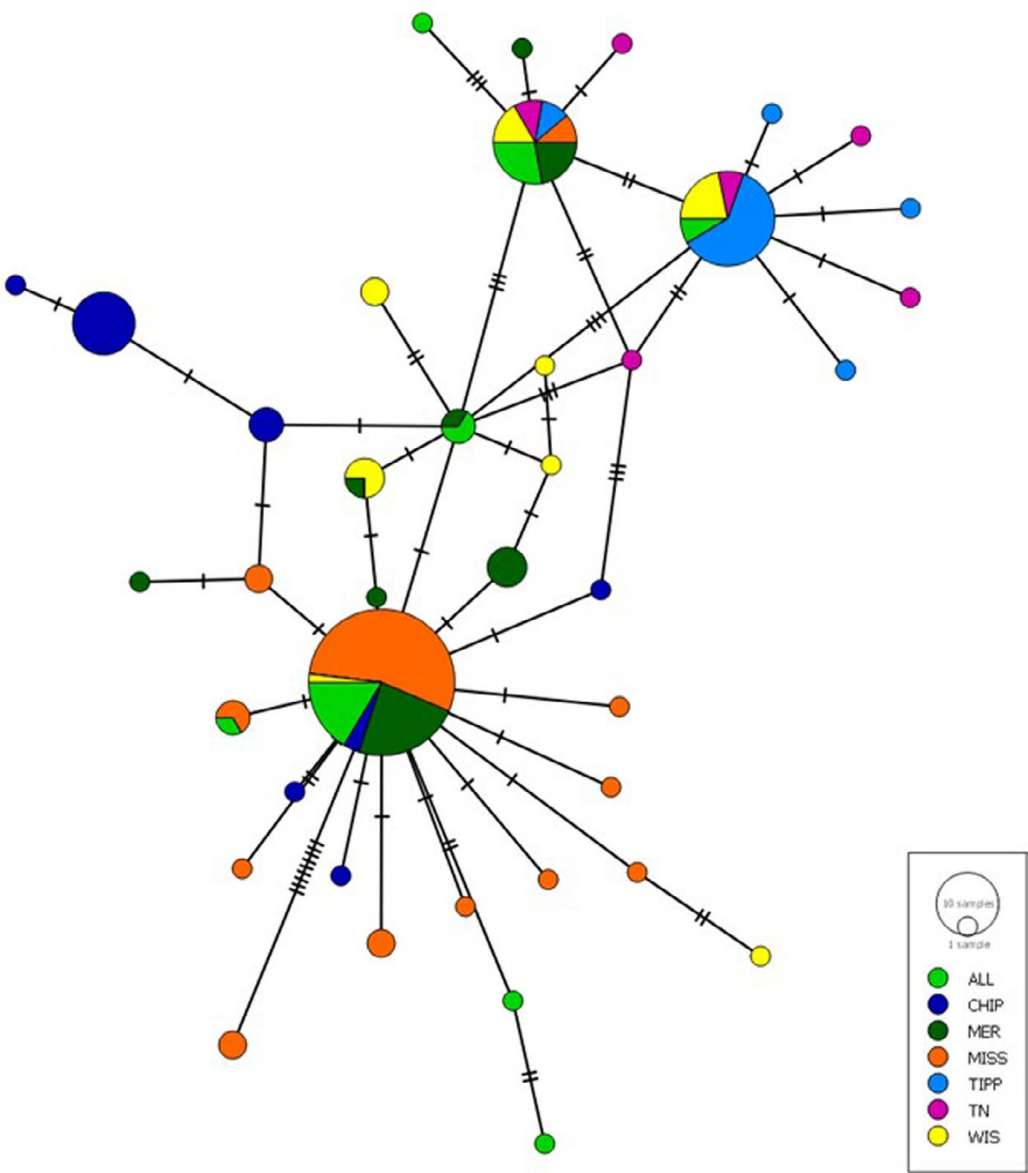
Minimum spanning network of 39 ND1 haplotypes. Colors indicate sites and circle size represents number of haplotypes. Circles composed of multiple colors indicate shared haplotypes. Tick marks indicate number of additional differences between haplotypes

Analyses were performed on 157 aligned DNA sequences of 883 nucleotide base pairs that had no missing data or ambiguous sites. Thirty‐nine mtDNA haplotypes were detected across all sample sites. The number of haplotypes ranged from 5 to 12 per sampling site (Table 4) with the MISS site having the most (*n* = 12) and the TIPP site having the fewest (n = 5). The number of unique haplotypes ranged from 3 to 9 per site and accounted for about 85% of detected haplotypes overall (33 of 39). Haplotype diversity (*H*_d_) ranged from 0.4620 (TIPP) to 0.9286 (TN) and the nucleotide diversity (π) ranged from 0.0008 (TIPP) to 0.0039 (WIS). A comparison of pairwise *F*_ST_ values indicated that all sample sites were significantly different from each other except the TIPP and ALL sites and the WIS and ALL sites (Table 5). A minimum spanning network indicated that only three haplotypes were shared across most sampling sites with a star‐like pattern stemming from these common haplotypes consistent with population expansion (Slatkin & Hudson, 1991) (Figure 4).

**TABLE 4:** Number of samples, haplotypes, unique haplotypes, haplotype diversity (*H*_d_), and nucleotide diversity (*π*) across all sites

Site ID	Samples	Number of haplotypes	Number of unique haplotypes	Haplotype Diversity (*H*_d_)	Nucleotide Diversity (*π*)	Sum of Square Deviations (SSD)	Raggedness Index
ALL	22	8	3	0.7922	0.0034	0.045 (0.19)	0.124 (0.14)
C.I. (95%)				(0.7235, 0.8609)	(0.0013, 0.0054)		
CHIP	19	7	6	0.7135	0.0020	0.025 (0.55)	0.079 (0.61)
C.I. (95%)				(0.6110, 0.8160)	(0.0006, 0.0033)		
MER	26	8	4	0.7231	0.0023	0.021 (0.68)	0.058 (0.73)
C.I. (95%)				(0.6424, 0.8038)	(0.0008, 0.0038)		
MISS	46	12	9	0.5749	0.0019	0.014 (0.12)	0.083 (0.56)
C.I. (95%)				(0.4878, 0.6620)	(0.0006, 0.0031)		
TIPP	19	5	3	0.4620	0.0008	0.004 (0.81)	0.112 (0.64)
C.I. (95%)				(0.3262, 0.5978)	(0.0001, 0.0015)		
TN	8	6	4	0.9286	0.0024	‐	‐
C.I. (95%)				(0.8442, 1.013)	(0.0007, 0.0041)		
WIS	17	8	4	0.8750	0.0039	0.044 (0.01*)	0.129 (0.02*)
C.I. (95%)				(0.8223, 0.9277)	(0.0016, 0.0063)		
*Total*	*157*	*39*	*33*	*0.7242*	*0.0024*		

Note

95% confidence intervals are given for the haplotype and nucleotide diversity values. Sum of Square Deviations (SSD) and Raggedness Index results of the mismatch distribution analysis across all sites. Values and associated p‐values are listed.

*Indicates significance.

**TABLE 5:** Pairwise *F*_ST_ values of mtDNA sequences

	ALL	CHIP	MER	MISS	TIPP	TN
ALL						
CHIP	0.2921*					
MER	0.4721*	0.1543*				
MISS	0.3370*	0.6357*	0.7506*			
TIPP	0.0268	0.5008*	0.6396*	0.3669*		
TN	0.1666*	0.6401*	0.7172*	0.4299*	0.0627*	
WIS	0.0607	0.3146*	0.3146*	0.3557*	0.1748*	0.3588*

Note

*Indicates significant differentiation at a 0.05 significance level.

#### 3.2.2 Population expansion

Evidence for a population expansion was found for all sites except WIS. The sum of square deviations and raggedness index indicated that these sites did not significantly differ from the population expansion model (*p* ≥ .05) (Table 4). The detected population expansion is also indicated by the star‐like pattern seen in the minimum spanning network (Slatkin & Hudson, 1991) (Figure 4). However, the WIS site indicated a significant deviation from the population expansion model in both the sum of square deviations (0.044, *p* = .01) and raggedness index (0.129, *p* = .02) (Table 4). Convergence could not be reached for the TN site, so it was dropped from the mismatch distribution analysis.

## 4 DISCUSSION

This study found that despite the lack of gene flow and population isolation, there is still a high degree of genetic diversity among sampling sites at both the microsatellite and mitochondrial loci. Similar levels of genetic diversity have been found in other studies of both rare and common freshwater mussel species (Elderkin et al., 2007; Geist & Kuehn, 2005; Inoue et al., 2014; King et al., 1999; Zanatta & Murphy, 2007, 2008), possibly indicating abilities of some freshwater mussels to maintain genetic diversity despite isolation. Isolated Sheepnose populations may be large enough to maintain high level of genetic diversity and buffer populations against the erosive effects of genetic drift (Elderkin et al., 2007; Lande & Barrowclough, 1987). Additionally, Sheepnose are estimated to have lifespans of up to 30 years (Hove et al., 2015), such long‐lived species can also buffer populations from the loss of genetic diversity due to drift (Hoffman et al., 2017). If this is true, efforts and resources aimed at conservation strategies such as propagation and translocations would be better directed toward regaining habitat suitability and connectivity (Olson & Vaughn, 2020).

Analysis of genetic structure indicates that currently, the Sheepnose consists of five genetically distinct populations. In the Mississippi River Basin, the populations consist of the Chippewa and the Wisconsin rivers, the Meramec River, and the Mississippi River. In the Ohio River Basin, the Allegheny River population is distinct from the Tennessee and Tippecanoe rivers. These populations are in turn composed of a number of distinct demes—isolated subpopulations that have a distinct gene pool. Both mitochondrial and microsatellite markers indicate a lack of connectivity between demes as evidenced by the unique mitochondrial haplotypes and private alleles and high values for pairwise *F*_ST_, adjusted *G*_ST_, and Jost's D (Tables 2 and 5). It is tempting to attribute the genetic differentiation detected between Sheepnose demes to be the result of habitat degradation that has fragmented the species range and isolated these groups from each other. Dams are considered highly detrimental to unionoid populations because they disrupt dispersal of host fishes (Watters, 1995), feeding ability (Bates, 1962; Negus, 1966), and alter stream flow and depth (Salmon & Green, 1983). The United States has about 75,000 dams and almost half (~30,000) can be found in the Mississippi River system (Graf, 1999). The direct impacts of dams on mussel health and their indirect impacts on dispersal may be contributing factors to the isolation between Sheepnose demes detected at the contemporary timescale. The construction of dams, river channelization, increased pollution, and invasive species are collectively contributing to the loss of habitat and changes in the distribution of freshwater mussels (Williams et al., 1989). However, the contemporary fragmentation of populations and demes due to anthropogenic barriers and habitat loss are not likely what is being detected in the analysis of the data for the Sheepnose. For long‐lived species with long generation times, and large *N_e_* like most unionids, it would most likely take centuries for any genetic signature of these anthropogenic issues to be detected using microsatellites (Haag, 2012; Hoffman et al., 2017). Instead, we propose that the differentiation observed is the result of changes in climate and on the landscape that occurred during and subsequent to the Pleistocene and before European contact in North America. The high number of private alleles and unique haplotypes found at each sample location indicate that these locations have been isolated for a substantial amount of time. Similar results have been found in other studies of freshwater mussels (Elderkin et al., 2007; Hewitt, 1996; Inoue et al., 2014; Jones et al., 2015; Tomilova et al., 2020). Together these studies indicate that for many freshwater mussels, population genetic structure is more reflective of long‐term factors related to changes since the Pleistocene era and not from recent anthropogenic causes.

The amount of migration estimated between sampling locations was low overall and very low rates were estimated between the Ohio and Mississippi river basins (Table 3). In the Mississippi River Basin, the migration appears to be unidirectional—from tributaries to the Mississippi River, which could be due to several factors including dispersal via fishes (glochidia), sperm gene flow, or downstream displacement during floods. In the Ohio River Basin, the Allegheny River appears to be receiving migrants from the Tennessee and Tippecanoe rivers. This result is harder to interpret, as it seems unlikely that host fishes carrying glochidia larvae could effectively travel between these sites. These results in the Ohio basin may instead be due to the absence of samples from the mainstem Ohio River. Host fish vagility may also be an important factor on the population structure observed in the Sheepnose. Previous studies on freshwater mussels have invoked host fishes as contributing to or reinforcing the population structure (Chong & Roe, 2018; Zanatta & Wilson, 2011). The most recent information indicates that hosts of the Sheepnose include cyprinids (Hove et al., 2015) which may lack the dispersal capabilities of larger riverine fishes (Comte & Olden, 2018) and therefore contribute to the isolation between populations and demes.

Despite the detected isolation, high levels of diversity were still observed within in demes. With the exception of the WIS deme, the analysis of mitochondrial data revealed a pattern consistent with expanding populations. Such a pattern may be a result of responses of the Sheepnose to climate change oscillations during the Pleistocene (Alberdi et al., 2015). North American freshwater systems were heavily impacted by the expansion and contraction of Pleistocene glaciers, and the population structure of freshwater organisms often reflect these events (Berendzen et al., 2010; Inoue & Berg, 2017; Inoue et al., 2014; Jones et al., 2015; Mathias et al., 2016; Pielou, 2008). Since the retreat of glaciers following the last glacial maxima, aquatic species in the Upper Mississippi River Basin have had opportunity to expand their ranges northward into previously glaciated regions (Hewitt, 1996, 2000; Inoue et al., 2014; Pielou, 2008; Stewart & Lister, 2001).

Our results indicate that the contemporary pattern of low gene flow and isolation occurring among populations is the result of prehistoric changes to the landscape that have eliminated populations and introduced barriers to gene flow. Although anthropogenic influences may be too recent to explain the observed patterns, they may be reinforcing existing genetic differences. It appears that the long lifespan of the Sheepnose may be delaying the reduction in genetic diversity typically associated with isolation. However, if isolation persists, it is possible that genetic diversity in these demes will start to erode. Our results suggest that efforts should be made to reestablish gene flow among demes to support the maintenance of genetic diversity. For example, Sheepnose could be re‐established within basins to facilitate connectivity between demes and within populations. The isolation of mussels into demes makes them more sensitive to stochastic events (Fagan et al., 2005), however, managers should be cautious about disrupting any local adaptions that may have been acquired by these demes (Fitzpatrick et al., 2015; Lean et al., 2017). Propagation and re‐introduction operations for reestablishing Sheepnose within their historical ranges (Geist, 2010; Jones et al., 2006; Minckley, 1995) should aim to avoid disrupting localized adaptations; therefore, translocations between different populations are not recommended (Fitzpatrick et al., 2015; Lean et al., 2017).

Overall, the Sheepnose appear to have maintained a surprising amount of genetic diversity within populations despite the lack of connectivity detected among demes. Mechanisms that may be buffering the erosion of genetic diversity such as their long lifespan and potentially high effective population sizes may not continue to withstand the negative effects of prolonged isolation and result in the eventual disappearance of the Sheepnose from portions of its current range. To effectively conserve this species, managers should create objectives focused on reestablishing and maintaining enough suitable habitat so Sheepnose can naturally reestablish populations and maintain connectivity between demes. Improved connectivity can facilitate the maintenance of genetic diversity and adaptive potential in the face of climate change and other emerging stressors.

## CONFLICT OF INTEREST

The authors certify that they have no affiliations with or involvement in any organization or entity with any financial interest, or non‐financial interest in the subject matter or materials discussed in this manuscript.

## AUTHOR CONTRIBUTIONS

**Sara Schwarz:** Data curation (equal); Formal analysis (lead); Methodology (equal); Writing – original draft (lead); Writing – review & editing (equal). **Kevin Roe:** Conceptualization (lead); Funding acquisition (lead); Project administration (lead); Writing – review & editing (equal).

### OPEN RESEARCH BADGES

This article has earned an Open Data Badge for making publicly available the digitally‐shareable data necessary to reproduce the reported results. The data is available at https://doi.org/10.5061/dryad.gxd2547mm.

## Supporting information

Appendix S1

## Data Availability

Mitochondrial DNA sequence data have been submitted to GenBank (Accession Number: MH853483). Microsatellite genotypes have been submitted to DRYAD (https://doi.org/10.5061/dryad.gxd2547mm).

## References

[ece38630-bib-0001] Alberdi, A. , Gilbert, M. T. P. , Razgour, O. , Aizpurua, O. , Aihartza, J. , & Garin, I. (2015). Contrasting population‐level responses to Pleistocene climactic oscillations in an alpine bat revealed by complete mitochondrial genomes and evolutionary history inference. Journal of Biogeography, 42(9), 1689–1700.

[ece38630-bib-0002] Andersen, L. W. , Fog, K. , & Damgaard, C. (2004). Habitat fragmentation causes bottlenecks and inbreeding in the European tree frog (*Hyla arborea*). Proceedings of the Royal Society B: Biological Sciences, 271(1545), 1293. 10.1098/rspb.2004.2720 PMC1691722 15306354

[ece38630-bib-0003] Bandelt, H. , Forster, P. , & Rӧhl, A. (1999). Median‐joining networks for inferring intraspecific phylogenies. Molecular Biology and Evolution, 16(1), 37–48. 10.1093/oxfordjournals.molbev.a026036 10331250

[ece38630-bib-0004] Bates, J. M. (1962). Impact of impoundment on the mussel fauna of Kentucky Reservoir, Tennessee River. The American Midland Naturalist, 68, 232–236.

[ece38630-bib-0005] Berendzen, P. B. , Dugan, J. F. , & Gamble, T. (2010). Post‐glacial expansion into the paleozoic plateau: evidence of an ozarkian refugium for the Ozark minnow *Notropis nubilus* (Teleostei: Cypriniformes). Journal of Fish Biology, 77, 1114–1136. 21039494 10.1111/j.1095-8649.2010.02769.x

[ece38630-bib-0006] Berg, D. J. , Haag, W. R. , Guttman, S. I. , & Sickel, J. B. (1995). Mantle biopsy: a technique for non‐destructive tissue‐sampling of freshwater mussels. Journal of the North American Benthological Society, 14, 577–581. 10.2307/1467542

[ece38630-bib-0007] Bogan, A. E. (2008). Global diversity of freshwater mussels (Mollusca, Bivalvia) in freshwater. Hydrobiologia, 595, 139–147. 10.1007/s10750-007-9011-7

[ece38630-bib-0008] Chong, J. P. , & Roe, K. J. (2018). A comparison of genetic diversity and population structure of the endangered scaleshell mussel (*Leptodea leptodon*), the fragile papershell (*Lepodea fragilis*) and their host‐fish the freshwater drum (*Aplodinotus grunniens*). Conservation Genetics, 19, 425–437.

[ece38630-bib-0009] Comte, L. , & Olden, J. D. (2018). Fish dispersal in flowing waters: A synthesis of movement‐ and genetic‐based studies. Fish and Fisheries, 19, 1063–1077. 10.1111/faf.12312

[ece38630-bib-0010] Cornuet, J. M. , & Luikart, G. (1996). Description and power analysis of two tests for detecting recent population bottlenecks from allele frequency data. Genetics, 144, 2001–2014. 10.1093/genetics/144.4.2001 8978083 PMC1207747

[ece38630-bib-0011] Dudgeon, D. , Arthington, A. H. , Gessner, M. O. , Kawabata, Z. , Knowler, D. J. , Leveque, C. , Naiman, R. J. , Prieur‐Richard, A. , Soto, D. , Stiassny, M. L. J. , & Sullivan, C. A. (2005). Freshwater biodiversity: importance, threats, status and conservation challenges. The Cambridge Philosophical Society, 81(2), 163–182. 10.1017/S1464793105006950 16336747

[ece38630-bib-0012] Elderkin, C. L. , Christian, A. D. , Vaughn, C. C. , Metcalfe‐Smith, J. L. , & Berg, D. J. (2007). Population genetics of the freshwater mussel, *Amblema plicata* (Say 1817) (Bivalvia: Unionidae): Evidence of high dispersal and post‐ glacial colonization. Conservation Genetics, 8, 355–372. 10.1007/s10592-006-9175-0

[ece38630-bib-0013] Evanno, G. , Regnaut, S. , & Goudet, J. (2005). Detecting the number of clusters of individuals using the software STRUCTURE: a simulation study. Molecular Ecology, 14, 2611–2620. 10.1111/j.1365-294X.2005.02553.x 15969739

[ece38630-bib-0014] Excoffier, L. , & Lischer, H. E. L. (2010). Arlequin suite ver 3.5: A new series of programs to perform population genetics analysis under Linux and Windows. Molecular Ecology Resources, 10, 564–567. 21565059 10.1111/j.1755-0998.2010.02847.x

[ece38630-bib-0015] Fagan, W. , Aumann, C. , Kennedy, M. , & Unmack, P. (2005). Rarity, fragmentation and the scale dependence of extinction risk in desert fishes. Ecology, 86, 34–41. 10.1890/04-0491

[ece38630-bib-0016] Ferguson, C. D. , Blum, M. J. , Raymer, M. L. , Eackles, M. S. , & Krane, D. E. (2013). Population structure, multiple paternity, and long‐distance transport of spermatozoa in the freshwater mussel *Lampsilis cardium* (Bivalvia: Unionidae). Freshwater Science, 32(1), 267–282.

[ece38630-bib-0017] Fitzpatrick, S. W. , Gerberich, J. C. , Kronenberger, J. A. , Angeloni, L. M. , & Funk, W. C. (2015). Locally adapted traits maintained in the face of high gene flow. Ecology Letters, 18(1), 37–47. 10.1111/ele.12388 25363522

[ece38630-bib-0018] Francis, R. M. (2016). POPHELPER: an R package and web app to analyze and visualize population structure. Molecular Ecology Resources, 17(1), 27–32. 26850166 10.1111/1755-0998.12509

[ece38630-bib-0019] Frankel, O. H. , & Soulé, M. E. (1981). Conservation and evolution. Cambridge University Press.

[ece38630-bib-0020] Geist, J. (2010). Strategies for the conservation of endangered freshwater pearl mussels (*Margaritifera* L.): a synthesis of Conservation Genetics and Ecology. Hydrobiologia, 644(1), 69–88.

[ece38630-bib-0021] Geist, J. , & Kuehn, R. (2005). Genetic diversity and differentiation of central European freshwater pearl mussel (*Margaritifera* L.) populations: implications for conservation and management. Molecular Ecology, 14(2), 425–439. 15660935 10.1111/j.1365-294X.2004.02420.x

[ece38630-bib-0022] Gerlach, G. , Jueterbock, A. , Kraemer, P. , Depperman, J. , & Harmand, P. (2010). Calculations of population differentiation based on Gst and D: forget Gst not all of statistics! Molecular Ecology, 19, 3845–3852. 20735737 10.1111/j.1365-294X.2010.04784.x

[ece38630-bib-0023] Goudet, J. , & Thibaut, J. (2015). Package hierfstat. R package version 0.04‐22. http://www.r‐project.org

[ece38630-bib-0024] Graf, D. L. , & Cummings, K. S. (2007). Review of the systematics and global diversity of freshwater mussel species (Bivalvia: Unionoida). Journal of Molluscan Studies, 73, 291–314. 10.1093/mollus/eym029

[ece38630-bib-0025] Graf, W. L. (1999). Dam nation: A geographic census of American dams and their large‐scale hydrologic impacts. Water Resources Research, 35(4), 1305–1311. 10.1029/1999WR900016

[ece38630-bib-0026] Haag, W. R. (2012). North American freshwater mussels. Cambridge Press. 10.1111/brv.12028 23445204

[ece38630-bib-0027] Haag, W. R. , & Williams, J. D. (2014). Biodiversity on the brink: an assessment of conservation strategies for North American freshwater mussels. Hydrobiologia, 735, 45–60. 10.1007/s10750-013-1524-7

[ece38630-bib-0028] Harpending, H. C. (1994). Signature of ancient population growth in a low‐resolution mitochondrial DNA mismatch distribution. Human Biology, 66(4), 591–600. 8088750

[ece38630-bib-0029] Harpending, H. C. , Sherry, S. T. , Rogers, A. R. , & Stoneking, M. (1993). Genetic structure of ancient human populations. Current Anthropology, 34(4), 483–496.

[ece38630-bib-0030] Hedrick, P. W. (2005). A standardized genetic differentiation measure. Evolution, 59(8), 1633–1638. 10.1111/j.0014-3820.2005.tb01814.x 16329237

[ece38630-bib-0031] Henley, W. F. , Grobler, P. J. , & Neves, R. J. (2006). Non‐invasive method to obtain DNA from freshwater mussels (Bivalvia: Unionidae). Journal of Shellfish Research, 25, 975–977.

[ece38630-bib-0032] Hewitt, G. (1996). Some genetic consequences of ice ages, and their role in divergence and speciation. Biological Journal of the Linnean Society, 58(3), 247–276. 10.1006/bijl.1996.0035

[ece38630-bib-0033] Hewitt, G. (2000). The genetic legacy of the Quaternary ice ages. Nature, 405, 907–913. 10.1038/35016000 10879524

[ece38630-bib-0034] Hoffman, J. R. , Willoughby, J. R. , Swanson, B. J. , Pangle, K. L. , & Zanatta, D. T. (2017). Detection of barriers to dispersal is masked by long lifespans and large population sizes. Ecology and Evolution, 7, 9613–9623. 10.1002/ece3.3470 29187994 PMC5696434

[ece38630-bib-0035] Hove, M. C. , Sietman, B. E. , Berg, M. S. , Frost, E. C. , Wolf, K. , Brady, T. R. , Boyer, S. L. , & Hornbach, D. J. (2015). Early life history of the sheepnose (*Plethobasus cyphyus*) (Mollusca: Bivalvia: Unionoida). Journal of Natural History, 50(9–10), 523–542.

[ece38630-bib-0036] Hulce, D. , Li, X. , & Snyder‐Leiby, T. (2011). GeneMarker^®^ Genotyping Software: Tools to Increase the Statistical Power of DNA Fragment Analysis. Journal of Biomolecular Techniques, 22(Supp), S35–S36.

[ece38630-bib-0037] Inoue, K. , & Berg, D. J. (2017). Predicting the effects of climate change on population connectivity and genetic diversity of an imperiled freshwater mussel, *Cumberlandia monodonta* (Bivalvia: Margaritiferidae), in riverine systems. Global Change Biology, 23(1), 94–107. 27225328 10.1111/gcb.13369

[ece38630-bib-0038] Inoue, K. , Monroe, E. M. , Elderkin, C. L. , & Berg, D. J. (2014). Phylogeographic and population genetic analyses reveal Pleistocene isolation followed by high gene flow in a wide ranging, but endangered, freshwater mussel. Heredity, 112, 282–290. 10.1038/hdy.2013.104 24149656 PMC3931176

[ece38630-bib-0039] Jones, J. W. , Hallerman, E. M. , & Neves, R. J. (2006). Genetic management guidelines for captive propagation of freshwater mussels (Unionidae). Journal of Shellfish Research, 25(2), 527–535.

[ece38630-bib-0040] Jones, J. W. , Neves, R. J. , & Hallerman, E. M. (2015). Historical demography of freshwater mussels (Bivalvia: Unionidae): genetic evidence for population expansion and contraction during the late Pleistocene and Holocene. Biological Journal of the Linnean Society, 114(2), 376–397. 10.1111/bij.12437

[ece38630-bib-0041] Jost, L. (2008). G_ST_ and its relatives do not measure differentiation. Molecular Ecology, 17(18), 4015–4026. 19238703 10.1111/j.1365-294x.2008.03887.x

[ece38630-bib-0042] Kamvar, Z. N. , Tabima, J. F. , & Grünwald, N. J. (2014). Poppr: an R package for genetic analysis of populations with clonal, partially, clonal, and/or sexual reproduction. Peer J, 2, e281. 24688859 10.7717/peerj.281 PMC3961149

[ece38630-bib-0043] Kearse, M. , Moir, R. , Wilson, A. , Stones‐Havas, S. , Cheung, M. , Sturrock, S. , Buxton, S. , Cooper, A. , Markowitz, S. , Duran, C. , Thierer, T. , Ashton, B. , Mentjies, P. , & Drummond, A. (2012). Geneious Basic: an integrated and extendable desktop software platform for the organization and analysis of sequence data. Bioinformatics, 28(12), 1647–1649. 10.1093/bioinformatics/bts199 22543367 PMC3371832

[ece38630-bib-0044] King, T. L. , Eackles, M. S. , Gjetvaj, B. , & Hoeh, W. R. (1999). Intraspecific phylogeography of *Lasmigona subviridis* (Bivalvia: Unionidae): Conservation implications of range discontinuity. Molecular Ecology, 8, S65–S78. 10.1046/j.1365-294X.1999.00784.x 10703552

[ece38630-bib-0045] Lande, R. , & Barrowclough, G. F. (1987). Effective population size, genetic variation, and their use in population management. Viable Populations for Conservation, 87, 124.

[ece38630-bib-0046] Lean, J. , Hammer, M. P. , Unmack, P. J. , Adams, M. , & Beheregaray, L. B. (2017). Landscape genetics informs mesohabitat preference and conservation priorities for a surrogate indicator species in a highly fragmented river system. Heredity, 118, 374–384. 10.1038/hdy.2016.111 27876805 PMC5345605

[ece38630-bib-0047] Leigh, J. W. , & Bryant, D. (2015). PopART: Full‐feature software for haplotype network construction. Methods in Ecology and Evolution, 6(9), 1110–1116.

[ece38630-bib-0048] Librado, P. , & Rozas, J. (2009). DnaSP v5: a software for comprehensive analysis of DNA polymorphism data. Bioinformatics, 25(11), 1451–1452. 10.1093/bioinformatics/btp187 19346325

[ece38630-bib-0049] Luikart, G. , Allendorf, F. W. , Sherwin, B. , & Cornuet, J. M. (1998). Distortion of allele frequency distributions provides a test for recent population bottlenecks. Journal of Heredity, 12, 238–247. 10.1093/jhered/89.3.238 9656466

[ece38630-bib-0050] Mathias, P. T. , Hoffman, J. R. , Wilson, C. C. , & Zanatta, D. T. (2016). Signature of postglacial colonization on contemporary genetic structure and diversity of *Quadrula* (Bivalvia: Unionidae). Hydrobiologia, 810, 207–225.

[ece38630-bib-0051] Minckley, W. L. (1995). Translocation as a tool for conserving imperiled fishes: Experiences in western United States. Biological Conservation, 72, 297–309. 10.1016/0006-3207(94)00091-4

[ece38630-bib-0052] Mutvei, H. , Westmark, T. , Dunca, E. , Carell, B. , Forberg, S. , & Bignert, A. (1994). Methods for the study of environmental changes using the structural and chemical information in molluscan shells. Past and present biomineralization processes, considerations about the carbonate cycle. Bulletin Du Musée Océanographique De Monaco, 13, 163–191.

[ece38630-bib-0053] Negus, J. K. (1966). A quantitative study of growth and reproduction of unionid mussels in the Thames River at Reading. Journal of Animal Ecology, 35, 513–532.

[ece38630-bib-0054] Oesch, R. D. (1984). Missouri Naiades: A guide to the mussels of Missouri (pp. 188–121). Missouri Department of Conservation.

[ece38630-bib-0055] Olson, J. P. , & Vaughn, C. C. (2020). Population Genetics of a Common Freshwater Mussel, *Amblema plicata*, in a Southern U.S. River. Freshwater Mollusk Biology and Conservation, 23(2), 124–133.

[ece38630-bib-0056] Parmalee, P. W. , & Bogan, A. E. (1998). The freshwater mussels of Tennessee (p. 328). The University of Tennessee Press.

[ece38630-bib-0057] Peakall, R. , & Smouse, P. E. (2012). GenAlEx 6.5: genetic analysis in Excel. Population genetic software for teaching and research‐an update. Bioinformatics, 28, 2537–2539. 10.1093/bioinformatics/bts460 22820204 PMC3463245

[ece38630-bib-0058] Pielou, E. C. (2008). After the ice age: The return of life to glaciated North America. University of Chicago Press.

[ece38630-bib-0059] Piry, S. , Luikart, G. , & Cornuet, J. M. (1999). BOTTLENECK: A computer program for detecting recent reductions in the effective population size using allele frequency data. Journal of Heredity, 90, 502–503.

[ece38630-bib-0060] Pritchard, J. K. , Stephens, M. , & Donnelly, P. (2000). Inference of population structure using multilocus genotype data. Genetics, 155(2), 945–959. 10.1093/genetics/155.2.945 10835412 PMC1461096

[ece38630-bib-0061] Rambaut, A. , Drummond, A. J. , Xie, D. , Baele, G. , & Suchard, M. A. (2018). Tracer v1.7. http://beast.community/tracer 10.1093/sysbio/syy032 PMC6101584 29718447

[ece38630-bib-0062] RStudio Team (2016). RStudio: Integrated Development for R. RStudio Inc. http://www.rstudio.com/

[ece38630-bib-0063] Salmon, A. , & Green, R. H. (1983). Environmental determinants of unionid clam distribution in the Middle Thames River, Ontario. Canadian Journal of Zoology, 61, 832–838. 10.1139/z83-109

[ece38630-bib-0064] Schuelke, M. (2000). An economic method for the fluorescent labeling of PCR fragments. Nature Biotechnology, 18, 233–234. 10.1038/72708 10657137

[ece38630-bib-0065] Serb, J. M. , Buhay, J. E. , & Lydeard, C. (2003). Molecular systematics of the North American freshwater bivalve genus *Quadrula* (Unionidae: Ambleminae) based on mitochondrial ND1 sequences. Molecular Phylogenetics and Evolution, 28(1), 1–11. 10.1016/S1055-7903(03)00026-5 12801467

[ece38630-bib-0066] Slatkin, M. , & Hudson, R. R. (1991). Pairwise comparisons of mitochondrial DNA sequences in stable and exponentially growing populations. Genetics, 129, 555–562. 10.1093/genetics/129.2.555 1743491 PMC1204643

[ece38630-bib-0067] Spiegelhalter, D. J. , Best, N. G. , Carlin, B. P. , & van der Linde, A. (2002). Bayesian measures of model complexity and fit. Journal of the Royal Statistical Society: Series B (Statistical Methodology), 64, 583–639. 10.1111/1467-9868.00353

[ece38630-bib-0068] Stein, B. A. , & Flack, S. R. (1997). Species report card: The state of US Plants and Animals. The Nature Conservancy.

[ece38630-bib-0069] Stewart, J. R. , & Lister, A. M. (2001). Cryptic northern refugia and the origins of modern biota. Trends in Ecology & Evolution, 16(11), 608–613.

[ece38630-bib-0070] Strayer, D. L. , & Dudgeon, D. (2010). Freshwater biodiversity conservation: recent progress and future challenges. Journal of the North American Benthological Society, 29(1), 344–358.

[ece38630-bib-0071] Surber, T. (1913). Notes on the natural hosts of fresh‐water mussels. US Government Printing Office.

[ece38630-bib-0072] Tomilova, A. A. , Lyubas, A. A. , Kondakov, A. V. , Vikhrev, I. V. , Gofarov, M. Y. , Kolosova, Y. S. , Vinarski, M. V. , Palatov, D. M. , & Bolotov, I. N. (2020). Evidence for Plio‐Pleistocene Duck Mussel Refugia in the Azvov Sea River Basins. Diversity, 12(3), 118.

[ece38630-bib-0073] Turgeon, D. D. , Quinn, J. F. , Bogan, A. E. , Coan, E. V. , Hochberg, F. G. , Lyons, W. G. , Mikkelsen, P. M. , Neves, R. J. , Roper, C. F. , Rosenburg, G. , & Roth, B. (1998). Common and scientific names of aquatic invertebrates from the United States and Canada: Mollusks. American Fisheries Society.

[ece38630-bib-0074] United States Army Corps of Engineers (2011). Biological Assessment for the federally endangered Pink Mucket (*Lampsilis abrupta*), Orangefoot Pimpleback (*Plethobasus cooperianus*), and threatened Snail Darter (*Percina tanasi*). United States Army Corps of Engineers.

[ece38630-bib-0075] United States Fish and Wildlife Service (1984). Recovery plan for the orange‐footed pearly mussel, *Plethobasus cooperianus* (Lea, 1834). United States Fish and Wildlife Service Region 4.

[ece38630-bib-0076] United States Fish and Wildlife Service (2002). Status Assessment Report for the sheepnose, *Plethobasus cyphyus*, occurring in the Mississippi River system. U.S. Fish and Wildlife Service regions 3, 4, 5.

[ece38630-bib-0077] United States Fish and Wildlife Service (2012a) Sheepnose Fact Sheet. http://www.fws.gov

[ece38630-bib-0078] United States Fish and Wildlife Service (2012b). Endangered and threatened wildlife and plants; Determination of endangered status for the sheepnose and spectaclecase mussels throughout their range. Federal Register, 77(49), 14914–14949.

[ece38630-bib-0079] Van Oosterhout, C. , Hutchinson, W. F. , Wills, D. P. , & Shipley, P. (2004). MICRO‐CHECKER: software for identifying and correcting genotyping errors in microsatellite data. Molecular Ecology Resources, 4(3), 535–538. 10.1111/j.1471-8286.2004.00684.x

[ece38630-bib-0080] Vaughn, C. C. (2018). Ecosystem services provided by freshwater mussels. Hydrobiolgia, 810, 15–27. 10.1007/s10750-017-3139-x

[ece38630-bib-0081] Vaughn, C. C. , Nichols, S. J. , & Spooner, D. E. (2008). Community and food web ecology of freshwater mussels. . Journal of the North American Benthological Society, 27, 409–423. 10.1899/07-058.1

[ece38630-bib-0082] Watters, G. T. (1995). Small dams as barriers to freshwater mussels (Bivalvia, Unionoida) and their hosts. Biological Conservation, 75, 79–85. 10.1016/0006-3207(95)00034-8

[ece38630-bib-0083] Williams, J. E. , Johnson, J. E. , Hendrickson, D. A. , Contreras‐Balderas, W. , Williams, J. D. , Navarro‐Mendoza, M. , McAllister, D. E. , & Deacon, J. E. (1989). Fishes of North America endangered, threatened, or of special concern: 1989. Fisheries, 14(6), 2–20.

[ece38630-bib-0084] Williams, J. D. , Warren, M. L. Jr , Cummings, K. S. , Harris, J. L. , & Neves, R. J. (2011). Conservation Status of Freshwater Mussels of the United States and Canada. Fisheries, 18(9), 6–22.

[ece38630-bib-0085] Wilson, G. A. , & Rannala, B. (2003). Bayesian inference of recent migration rates using multilocus genotypes. Genetics, 163(3), 1177–1191. 12663554 10.1093/genetics/163.3.1177 PMC1462502

[ece38630-bib-0086] Wright, S. (1969). Evolution and the Genetics of Populations, Vol. 2. University of Chicago Press.

[ece38630-bib-0087] Zanatta, D. T. , & Murphy, R. W. (2007). Range‐wide population genetic analysis of the endangered northern riffleshell mussel, *Epioblasma torulosa rangiana* (Bivalvia: Unionoida). Conservation Genetics, 8, 1393–1404.

[ece38630-bib-0088] Zanatta, D. T. , & Murphy, R. W. (2008). The phylogeographical and management implications of genetic population structure in the imperiled snuffbox mussel, *Epioblasma triquetra* (Bivalvia: Unionidae). Biological Journal of the Linnean Society, 93, 371–384.

[ece38630-bib-0089] Zanatta, D. T. , & Wilson, C. C. (2011). Testing congruency of geographic and genetic population structure for a freshwater mussel (Bivalvia: Unionoida) and its host fish. Biological Journal of the Linnean Society, 103(3), 669–685.

