# Supplementary material for: Population structure and gene flow in the Sheepnose mussel (Plethobasus cyphyus) and their implications for conservation
Source: Ecol Evol. 2022 Feb 17;12(2):e8630. doi: 10.1002/ece3.8630 (PMC8854780; doi:10.1002/ece3.8630)
Supplement: Supplementary file 1 — Appendix S1 [file ECE3-12-e8630-s001.docx]

**Appendix 1.** List of loci with the associated repeat motif, primer sequence, annealing temperature, and number of alleles for each locus. * indicates values that were not reported because the locus was dropped from analysis.

| Locus | Repeat Motif | Primer Sequence | °C | # Alleles |
| --- | --- | --- | --- | --- |
| A103-F  A103-R | (CA)_N_ | cca acg tca ata agc tgt agt c  tta caa cac atg gca att act g | 55 | 22 |
| A115-F  A115-R | (CA)_N_ | tac cct ggc act caa ata ctt g  cct ggt gtc agg ata atg tga c | 57 | 25 |
| A120-F  A120-R | (CA)_N_ | agc aaa cag ttc ctc gtt c  tcc tcg tgt att gta gca aac | 55 | 31 |
| C1-F  C1-R | (TACA)_N_ | gag cgg tct gac tgg aac  acg aga gtt tgt gct cca c | 55 | 31 |
| C6-F  C6-R | (TACA)_N_ | gca gtg tat ggc aat gaa ca  gcg taa taa cct gtg acc tcc | 55 | 17 |
| C105-F  C105-R | (TACA)_N_ | ttg cat gtg tca ctt cat act g  gca cct acc tac cta tct ctc g | 55 | 32 |
| C109A-F  C109A-R | (TACA)_N_ | ggt atg gct tag atg cag ttc  act tgc aca cac gta agt acg | 59.4 | * |
| C115-F  C115-R | (TACA)_N_ | cat gga atg tac tgg tgt cg  agc tcg gct gtt aga aat tg | 59.4 | * |
| C125-F  C125-R | (TACA)_n_ | gga cgc tct aac cac tag gc  agt cca gat ttg att gct tca g | 57 | 17 |
| D4-F  D4-R | (TAGA)_n_ | cgg cct tta aga aga tcc c  tca ggc tga cca ccg tag | 55 | 47 |
| D10-F  D10-R | (TAGA)_n_ | tca aca tta ttc cag gct att c  tcg aca agg tca agt aca aag | 55 | 11 |
| D106-F  D106-R | (TAGA)_n_ | tct ctg tgt aac gga ttc tga g  tga agg gca aat cac ctc | 55 | 18 |
| D113-F  D113-R | (TAGA)_n_ | taa aag aag ctc cat cac atg  agt tgc atc agt tgt atg att g | 57 | * |
| D114-F  D114-R | (TAGA)_n_ | gtg ggt tct ttc tcc acc ag  gtc aag tgt gac tgc gtc aac | 55 | 14 |
| D119-F  D119-R | (TAGA)_n_ | aca ttc atc cca gca gtt ag  gac agc ata cat ttg gac ttg | 58 | 15 |
| D125-F  D125-R | (TAGA)_n_ | tgg atg gat aga tca atg gat  cac cag cag aaa tat aga ctg g | 55 | 15 |
|  |  |  |  |  |
